# Occupational Therapy and Physiotherapy in Acute Stroke: Do Rural Patients Receive Less Therapy?

**DOI:** 10.1155/2016/1582706

**Published:** 2016-09-26

**Authors:** Josie Merchant, Gemma Kitsos, Samantha Ashby, Alex Kitsos, Isobel J. Hubbard

**Affiliations:** ^1^Occupational Therapy, School of Health Sciences, University of Newcastle, Callaghan, NSW 2308, Australia; ^2^Neurology Department, Hunter New England Area Health Service, New Lambton Heights, NSW 2305, Australia; ^3^Hunter New England Area Health Service, New Lambton Heights, NSW 2305, Australia; ^4^School of Medicine and Public Health, University of Newcastle, Callaghan, NSW 2308, Australia

## Abstract

*Objective*. To assess whether acute stroke patients in rural hospitals receive less occupational therapy and physiotherapy than those in metropolitan hospitals.* Design*. Retrospective case-control study of health data in patients ≤10 days after stroke.* Setting*. Occupational therapy and physiotherapy services in four rural hospitals and one metropolitan hospital.* Participants*. Acute stroke patients admitted in one health district.* Main Outcome Measures*. Frequency and duration of face-to-face and indirect therapy sessions.* Results*. Rural hospitals admitted 363 patients and metropolitan hospital admitted 378 patients. Mean age was 73 years. Those in rural hospitals received more face-to-face (*p* > 0.0014) and indirect (*p* = 0.001) occupational therapy when compared to those in the metropolitan hospital. Face-to-face sessions lasted longer (*p* = 0.001). Patients admitted to the metropolitan hospital received more face-to-face (*p* > 0.000) and indirect (*p* > 0.000) physiotherapy when compared to those admitted to rural hospitals. Face-to-face sessions were shorter (*p* > 0.000). Almost all were seen within 24 hours of referral.* Conclusions*. Acute stroke patients in Australian rural hospital may receive more occupational therapy and less physiotherapy than those in metropolitan hospitals. The dose of therapy was lower than recommended, and the referral process may unnecessarily delay the time from admission to a patient's first therapy session.

## 1. Introduction

In Australia, stroke is a leading cause of mortality and morbidity, affecting one in six adults each year [1, 2]. Stroke can adversely impact a person's ability to participate in everyday activities and evidence indicates that the healthcare prescribed and applied in the first few days (acute) after stroke is significant when it comes to recovery outcomes [3, 4]. Allied health professionals, including occupational therapists and physiotherapists, are key contributors to the recovery process in stroke patients [5, 6].

The Stroke Foundation's nationally agreed clinical guidelines [7] recommend early, intensive rehabilitation following stroke to improve outcomes and adherence to the guidelines has resulted in measureable benefit to patients [8]. Healthcare that targets functional outcomes and promotes independence is central to poststroke occupational therapy (OT) and physiotherapy (PT) [9, 10]. OT focusses on sensorimotor function (particularly related to upper limb), participation in everyday activities, processing skills, and the adaptation of an occupation or environment [11, 12]. PT focusses on patterns of movement, cardiovascular resilience, mobility, balance, and gait [13]. Both professions assess a patient's baseline function, focus on managing risk related to falls, and hypothesise the most beneficial pathway of care [14–16].

In Australian hospitals, there is evidence that although intensive intervention is important to maximise poststroke recovery, patients with a recent stroke are often “alone and inactive for much of the day” [17]. Considering the additional challenges facing health services in rural Australia [18, 19], it could be easy to assume that acute stroke patients admitted to rural hospitals receive less OT and PT than those admitted to metropolitan hospitals [2]. This study will test this assumption in patients admitted who are less than 10 days after stroke and compare the OT and PT service delivery in one metropolitan hospital to the OT and PT in four rural hospitals.

## 2. Methodology

### 2.1. Design

This retrospective case-control study analysed OT and PT service delivery data in acute stroke across five participating hospitals. All participating hospitals were located in the same Local Health District in New South Wales, Australia. The metropolitan hospital was the reference (control) against which data from the four hospitals were combined and used as a single comparator (case) (Table 1). The metropolitan hospital is the referral hospital for the four rural hospitals. The rural hospitals include two that are classified as “regional” and two that are classified as “rural” as defined by the Australian Standard Geographical Classification: Remoteness [20]. For the purposes of this study, all four hospitals will be referred to as rural.

### 2.2. Procedure

This study received ethical approval from the region's NSW Health Human Research Ethics Committee and this provided access to data from the health services' hospital and allied health databases. Identifiable data were only accessible to the researchers and all data were deidentified prior to analysis. All databases used in this study were standardised across all five hospitals and all patient data were routinely collected. The allied health database provided data specific to OT and PT service delivery. All therapists were required to submit day-to-day data which included the number of sessions provided (frequency), the time spent in each session (duration), and whether a session was direct (face-to-face) or indirect. Study data were limited to sessions undertaken in the first 10 days after stroke.

**Figure 1:**
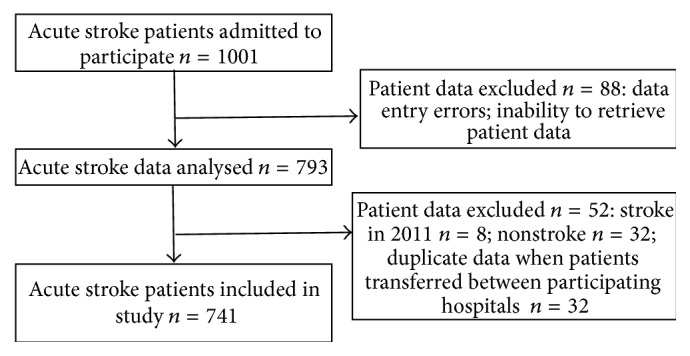
Study flow chart.

Data used in this study were recorded between January 1, 2012, and December 31, 2012. Patients excluded from the analysis were those with a secondary diagnosis of stroke on admission, those admitted for subacute care only, and/or those awaiting placement in supported accommodation (Figure 1). Patients were also excluded if they were admitted in late 2011 and were still in hospital during 2012, if they had no allied health data, and/or if they died during the 10-day period before receiving therapy. Additional patients were removed as they did not meet the inclusion criteria (Figure 1). This resulted in the analysis of data from 741 acute stroke patients across five hospitals.

### 2.3. Outcome Measures

The* primary* outcome was service delivery for OT and PT as measured in frequency and duration of sessions against type of session. The type of session was defined as direct or indirect. Direct sessions included face-to-face contact with the patient and their family and/or carers and phone calls and/or meetings where the patient and/or family and carers were present. Indirect therapy included report-writing, attending case conferences, and any discharge or session planning that did not involve direct contact with the patient and/or their family.

The* secondary* outcome was time of first contact with OT and PT. The Stroke Foundation's clinical guidelines recommend that patients should receive their first appointment within 24 hours of admission; this study investigated whether or not a patient was first seen by OT and PT within 24 hours of admission. Because some hospitals require that a medical referral be forwarded to therapists, this study also investigated whether or not the patient was seen within 24 hours of being referred.

### 2.4. Data Analysis

Data were analysed using STATA [21]. Descriptive statistics described the frequency of sessions, duration of session, and the type of session (direct or indirect). A* t*-test with *p* values set at 0.001 was used to test for differences between what occurred in the metropolitan hospital and what occurred in the rural hospitals.

## 3. Results

In 2012, the rural hospitals admitted 363 patients with stroke and the metropolitan hospital admitted 378 patients (*n* = 741) who met the study inclusion criteria.

Patients received 7686 sessions from OT and PT combined: 2023 (39%) were OT and 4663 (61%) were PT, and these were evenly distributed between the rural hospitals (*n* = 3523; 46%) and the metropolitan hospital (*n* = 4163; 54%).

### 3.1. OT and PT: Do Rural Patients Receive Less Therapy? 

Occupational therapists provided more sessions overall to patients with a recent stroke in the rural hospitals when compared to their colleagues in the metropolitan hospital and the face-to-face (direct) sessions lasted longer. Physiotherapists provided more sessions to patients in the metropolitan hospital but the face-to-face sessions lasted longer if the patient was admitted to a rural hospital (Table 3).

For OT, when compared to their counterparts in the metropolitan hospital, therapists provided more face-to-face (*p* > 0.0014) and indirect sessions (*p* = 0.001) to patients admitted to the rural hospitals. On average, rural patients received 1.4 face-to-face sessions and these lasted longer (*p* = 0.001) than the average 0.7 sessions that patients in the metropolitan hospital received. In respect to indirect OT, patients in the rural hospitals received 2.3 sessions compared to 1.6 sessions in the metropolitan hospital, but there was no difference in the duration of these sessions. Irrespective of whether or not the hospital was rural or metropolitan, face-to-face sessions were around 35 to 40 minutes long and indirect sessions were around 15 minutes. Two-thirds (64%) of all OT sessions were indirect.

In contrast, PT provided more face-to-face (*p* > 0.000) and indirect sessions (*p* > 0.000) to patients admitted to the metropolitan hospital when compared to sessions in the rural hospitals. On average, patients in the metropolitan hospital received 3 face-to-face sessions but they were shorter (*p* > 0.000) than the 2.5 sessions offered to patients in the rural hospitals. Irrespective of whether or not the hospital was rural or metropolitan, on average, all face-to-face sessions lasted 30 to 37 minutes and all indirect sessions were around 9 minutes long. In respect to indirect PT, patients in the metropolitan hospitals received 3 sessions compared to less than one session in the rural hospital, and these lasted longer in the metropolitan hospital. More than half (58%) of all PT sessions were face-to-face, but their frequency was highest (70%) in the rural hospitals.

Less than half of all patients (44%) had their first OT or PT session within 24 hours of their admission (Table 2), but almost all patients were seen within 24 hours of being referred to OT and PT, and both results were irrespective of whether the patient was admitted to a rural or metropolitan hospital.

## 4. Discussion 

This study found that, in the first 10 days after stroke, patients admitted to the rural hospitals received more OT than patients admitted to the metropolitan hospital but may have received less PT if they were admitted to a rural hospital. On average, all patients received between 1 and 3 face-to-face OT and/or PT sessions and these averaged between 30 and 40 minutes. The study found that when a patient with a recent stroke is referred to therapy may be more influential than when a patient is admitted to hospital. This is irrespective of whether or not a patient was admitted to the metropolitan hospital or one of the rural hospitals. This study finds that patients admitted to rural hospitals in Australia do not necessarily receive less therapy than their metropolitan counterparts; however, it provides evidence that the OT and PT service delivery does not consistently meet recommended nationally agreed, clinical guidelines.

### 4.1. Did Rural Patients Receive Less Occupational Therapy?

This study provides evidence that patients who have experienced a recent stroke and are admitted to rural hospitals can be receiving as much OT as, or* more* OT than, patients admitted to a large, city-based hospital. Not only were the rural patients in this study receiving more therapy sessions, but also these sessions were longer in duration. Although it is beyond the parameters of this study to assess whether or not the amount of the OT that patients received was optimal for recovery, it is reassuring to know that rural patients were not potentially disadvantaged in the amount of therapy they received. However, it is worrisome to know that patients with stroke received only two face-to-face OT sessions in the first 10 days after stroke and current recommendations are up to 1–3 hours a day and 3–5 times a week [22]. This study indicates that the OT patients receive needs clinical appraisal [7] and a revision in the current model of practice.

### 4.2. Did Rural Patients Receive Less Physiotherapy?

This study finds that patients who have experienced a recent stroke and are admitted to rural hospital can be receiving* less* frequent PT than patients admitted to a large, city-based hospital. However, these findings are not straightforward. Although patients in the metropolitan hospital received slightly more face-to-face sessions, these sessions were shorter in duration than the sessions that patients in rural hospitals received. In addition, when comparing face-to-face and indirect sessions, rural patients received a higher percentage of face-to-face PT. As with OT, this study cannot assess whether the amount PT is promoting recovery, but at best, receiving only three 30 to 35 minutes of face-to-face PT in the first 10 days after stroke is less than the recommended 1–3 hours a day, 3–5 times a week [22]. As with OT, clinical practice needs reviewing.

### 4.3. Is the Referral Process for OT and PT a Barrier to Timely Care?

For both OT and PT, this study found that a referral-to-first-appointment was twice as likely to be under 24 hours as admission-to-first-session. This finding highlights the clinical significance of the referral process in the allied healthcare provided to patients with acute stroke. Assuming that the referral process could take 24 hours to be enacted (and even longer if the referral was over a weekend), this raises questions as to whether a routine referral process is an unnecessary barrier to the key performance requirement that an allied health assessment be completed within 24–48 hours of admission following stroke [23].

### 4.4. Study Strengths and Limitations

This study's limitation includes the fact that the data were retrospective so there was no means by which data could be reviewed by therapists or those who make the data entries. However, this is also one of its strengths as the raw data cannot be manipulated or resubmitted at a later date. It was not possible to report on the OT and PT staffing levels across the participating hospitals and to factor this into the analysis. The data were limited to only one Local Health District in NSW so caution is required before generalising these findings across other hospitals in Australia and beyond.

## 5. Conclusion

This study found that patients admitted to rural hospitals with a recent stroke were not necessarily receiving less OT or PT than patients admitted to a metropolitan hospital. Even though there can be health inequities on the basis of geographic location, this study found that acute stroke patients admitted to rural hospitals may be receiving more OT but that the hospital's location may have little impact on how much PT patients receive. Consideration needs to be given to whether or not a referral process is unnecessarily delaying the time when patients with acute stroke receive their first OT and/or PT session. These findings have translational implications related to the current doses of OT and PT provided and applied to patients who are less than two weeks after stroke. The findings also challenge assumptions that rural patients may be receiving less therapy.

## Figures and Tables

**Table 1:** Characteristics of participating hospitals.

	Participating hospital	Stroke patients admitted (*n*)	Model of stroke care
Case	Regional 1	58	Stroke unit
	Regional 2	97	Medical ward
	Rural 1	104	Hub and spoke
	Rural 2	104	Hub and spoke

Total admissions		363	

Control	Metropolitan	378	Acute stroke unit

**Table 2:** Demographic data.

Occupational therapy and physiotherapy combined	Rural hospitals	Metropolitan hospital	Total	*p* value
Age in years: mean (SD)	75.2 (12.8)	71.3 (14.4)	73.2 (13.8)	0.0001^§^
Died < 10 d after stroke: *n* (%)	20 (5%)	28 (7%)	48 (6%)	0.2897
Seen < 24 h of admission: *n* (%)	151 (41.6%)	205 (54.4%)	323 (43.7%)	0.2697
Seen < 24 h of referral: *n* (%)	318 (87.9%)	351 (93.1%)	669 (90.5%)	0.0147

d: days; h: hours; §: significant.

**Table 3:** Comparing frequency and duration of OT and PT sessions against type of session and hospital/s.

Hospital/s	Rural (363)	Metropolitan (378)	Total (741)	*p* value
*Occupational therapy sessions*				
Frequency of direct sessions: *n* (%)	715 (38.2%)	374 (32.5%)	1089 (36%)	0.0014^§^
Direct sessions per patient: mean	1.46	0.74	1.47	
Frequency of indirect sessions: *n* (%)	1156 (61.8%)	778 (67.5%)	1934 (64%)	0.001^§^
Indirect sessions per patient: mean	2.32	1.55	2.61	
Direct sessions in minutes: mean (SD)	39.0 (20.3)	36.7 (20.9)	38.3 (20.5)	0.001^§^
Indirect sessions in minutes: mean (SD)	14.6 (8.8)	15.2 (11.5)	14.8 (10)	0.022

*Physiotherapy sessions*				
Frequency of direct sessions: *n* (%)	1207 (73.1%)	1489 (49.5%)	2696 (57.8%)	>0.000^§^
Direct sessions per patient: mean	2.42	2.96	3.64	
Frequency of indirect sessions: *n* (%)	445 (26.9%)	1522 (50.6%)	1967 (42.2%)	>0.000^§^
Indirect sessions per patient: mean	0.89	3.03	2.65	
Direct sessions in minutes: mean (SD)	37.1 (15.5)	30.7 (12.2)	33.5 (14.1)	>0.000^§^
Indirect sessions in minutes: mean (SD)	8.9 (4.6)	9.3 (5.3)	9.2 (5.1)	0.001^§^

SD: standard deviation; ^§^statistically significant.

## Additional Points

What Is Already Known on This Subject? (i) Rural-based patients diagnosed with recent stroke can be disadvantaged in the healthcare they receive when compared to metropolitan-based patients. (ii) Evidence indicates that it can be difficult to retain occupational therapists and physiotherapists in rural settings across Australia. (iii) Current nationally agreed guidelines recommend that acute stroke patients receive occupational therapy (OT) and physiotherapy (PT) up to 1–3 hours a day and 3–5 times a week. What Does This Study Add? (i) On average, acute stroke patient receives between 1 and 3 face-to-face OT and/or PT sessions and these average between 30 and 40 minutes in duration. (ii) The referral process to OT or PT for acute stroke patient may be unnecessarily delaying timely care after admission. (iii) In the first 10 days after stroke, rural-based patients can be disadvantaged in the healthcare they receive when compared to metropolitan-based patients.
